# Effects of the mHealth Supportive Care Program for Family Caregivers of Individuals With Dementia and Diabetes: Pilot Randomized Controlled Trial

**DOI:** 10.2196/72012

**Published:** 2026-03-24

**Authors:** Xinying Lin, Miaoqian Huang, Tuantuan Guo, Rong Lin, Chenshan Huang, Yuanjiao Yan, Hong Li

**Affiliations:** 1 The School of Nursing, Fujian Medical University, No.1, Xuefu North Road, Minhou County, Fuzhou, Fujian, 350122, China, 86 0591-22862504; 2 Xiangping Street Community Health Service Center, Tong'an District, XiaMen, China; 3 Department of General Practice, The Second Affiliated Hospital of Fujian Medical University, Quanzhou, China

**Keywords:** senile dementia, diabetes, family caregivers, mobile health, supportive care, randomized controlled trial

## Abstract

**Background:**

The comorbidity of dementia and type 2 diabetes mellitus exacerbates the burden on family caregivers (FCGs). Mobile health (mHealth) technology offers a promising alternative to overcome the spatiotemporal limitations of traditional interventions, but evidence for its efficacy in supporting dementia–type 2 diabetes mellitus caregivers remains scarce.

**Objective:**

This study aimed to evaluate the effectiveness of an mHealth supportive care program for FCGs of individuals with dementia and diabetes, focusing on caregiver burden, social support, and dementia care knowledge.

**Methods:**

A 2-arm, parallel-group randomized controlled trial was conducted. Between September 2022 and January 2023, FCGs were recruited from 5 urban and 10 rural communities under a community health center in Xiamen, China. Eligible caregivers were legally related to the patient, providing care for more than 8 hours per day for at least 1 month, conscious adults with basic literacy, owning and able to use a smartphone, and willing to provide informed consent. Their care recipients met diagnostic criteria for both dementia and type 2 diabetes, aged more than 60 years. Participants were randomly allocated (1:1) to intervention (n=30) or wait-list control (n=30). The intervention group received a 12-week mHealth supportive care program via the “Xiamen i-Health” platform, comprising 6 core modules (updated biweekly) and on-demand teleconsultation, in addition to conventional offline health education. The control group received conventional monthly 1-hour home-visit health education only. The primary outcome was caregiver burden measured by the Caregiver Burden Inventory (CBI). Secondary outcomes included social support (Social Support Rating Scale; SSRS) and dementia care knowledge (Dementia Care Knowledge Scale; DCKS). Assessments were performed at baseline (T0) and 3-month postintervention (T1). Only data collectors and statistical analysts were blinded.

**Results:**

Of 108 potential participants, 60 were randomly assigned. Per-protocol analysis included 55 participants (intervention group n=28 and control n=27). Postintervention, the intervention group showed a significantly greater reduction in CBI scores compared to the control group (between-group difference, *Z*=−3.534, *P*<.001, *r*=0.477). The intervention group also demonstrated significantly greater improvements in SSRS (*Z*=2.494, *P*=.01) and DCKS scores (*Z*=−4.233, *P*<.001, *r*=0.570). Subgroup analyses revealed that the reduction in caregiver burden was more pronounced among male, younger (60 y), physical labor, and lower-income caregivers. No intervention-related adverse events were reported.

**Conclusions:**

This theoretically grounded mHealth supportive care program effectively reduced burden and improved outcomes for FCGs managing dementia-diabetes comorbidity. The integrated online-offline delivery model shows particular promise for male, younger, manual labor, and lower-income caregivers, suggesting mHealth's potential to address health equity. These findings provide a feasible model for scalable caregiver support in resource-limited primary care settings. Future research should involve multicenter trials with longer follow-up and cost-effectiveness analyses.

## Introduction

Dementia care remains a significant public health challenge for health systems worldwide. By 2019, the economic cost of dementia had escalated to $1.3 trillion globally [1], posing a substantial financial strain. In China, the older population (age ≥60 y) living with dementia has reached 15.07 million, with incidence rates increasing markedly with age [2]. While pharmacological treatments delay disease progression to some extent, the absence of definitive curative measures leads to patients progressively losing their self-care, work, and social abilities [3]. This dramatically increases the demand for dementia care, requiring round-the-clock care for advanced disease [4]. Notably, in China, approximately 70% of patients with dementia rely on family caregivers (FCGs), who bear a disproportionately heavy burden and face numerous challenges. The caregiving challenge becomes markedly more complex when dementia coexists with type 2 diabetes mellitus (T2DM). Epidemiological studies demonstrate that T2DM elevates Alzheimer disease risk by 1.5 to 2 times [5], driven by shared pathophysiology including insulin resistance and oxidative stress that accelerate cognitive decline [6]. The progression from mild cognitive impairment to dementia occurs in 6% to 25% of patients with T2DM, markedly higher than the 0.2% to 3.9% observed in age-matched controls [5]. This comorbidity leads to compounded cognitive deterioration [7] and severely impairs self-care management [8] and long-term quality of life [9]. Patients often struggle with medication adherence, frequently forgetting or duplicating doses, which results in dangerous glycemic fluctuations that may further exacerbate cognitive dysfunction [10]. China’s metabolic disease epidemic further amplifies this issue, with over 140.9 million diabetes cases [11], 95% being T2DM [12]. The clinical complexity of dementia-T2DM comorbidity generates substantially greater caregiver burdens, as FCGs must concurrently manage dementia-related behavioral symptoms and diabetes-specific care requirements, including meticulous medication supervision, dietary control, and hypoglycemia prevention. These patients exhibit highly variable symptomatology [13,14] and extremely high care dependency [15], yet existing research predominantly focuses on patient self-management rather than critical external determinants like social support, caregiving contexts, or health care resource availability [16-18].

As a vital extension of the health care system, FCGs play an indispensable role in caring for patients with dementia and diabetes [19,20]. Enhancing FCG care skills and addressing their care needs are pivotal to mitigating public health pressures and improving patient quality of life. In developing countries, the paucity of primary health care resources shifts a greater proportion of caregiving responsibilities to FCGs, thereby augmenting their burden. While FCGs, as unpaid caregivers [21], may derive emotional fulfillment and personal growth from caregiving [22], they face numerous challenges, including psychological, emotional, physical, and financial burdens [23], which often lead to social isolation and reduced quality of life and physical health [24-27]. In China, strong family ties and a profound sense of responsibility socially obligate family members to undertake caregiving duties, placing an emotional burden on them when caring for frail relatives [28]. Studies indicate that caregivers are more likely to choose institutionalization when experiencing high emotional stress and burden [29], and those under greater stress face a 63% higher mortality rate compared to others [30]. Consequently, caregivers are frequently referred to as “hidden patients” [31].

Furthermore, as FCGs are often instructed to prioritize managing patients’ symptoms [32], their needs are frequently overlooked and unacknowledged by health and social care professionals [33]. Patients with dementia and diabetes require intricate and meticulous care due to cognitive decline and metabolic abnormalities, increasing caregiving challenges and placing higher demands on FCGs. According to a survey by the Alzheimer’s Disease Society of China, the primary challenges faced by the FCGs of patients with dementia include inadequate caregiving skills, limited care resources, and restricted treatment services. The level of care often exceeds the FCGs’ capabilities, potentially increasing their burden and worsening mental health issues [34]. Developing effective strategies to support caregiving is imperative. Targeted interventions to train and support FCGs can enhance their competence [35], reducing caregiver stress and burden.

Social support and caregiving knowledge are critical for dementia-combined diabetes caregivers. Studies have shown that educational interventions can significantly reduce caregiver anxiety and burden and improve patient quality of life [36,37], while unmet information needs can lead to reduced caregiving outcomes [38]. Werner et al [39] noted that caregivers need timely, personalized, and accessible information support. Effective interventions (eg, providing education and practical information) can improve knowledge, reduce stress, and enhance caregiving [40-42]. In addition, caregiver needs span emotional social support, knowledge and skills training, and self-management [43], but existing resources often lack credibility or have mismatched learning styles [44]. Digital support tools (eg, teleconsultation) can provide flexible and professional guidance that can improve caregiver mental health [45] and reduce caregiver stress and burden [46-48], and multicomponent interventions are particularly effective [49,50].

The World Health Organization emphasizes the importance of supporting families caring for older adults in its guidelines for community-based integrated care [51]. Advancements in mobile health (mHealth) technology, which overcome time and space constraints and expand the intervention scope, have led to its widespread application in chronic disease management and home care [52,53]. Internet-based supportive interventions have been implemented in countries such as the United Kingdom [54], Germany [55], and India [56], offering convenient and effective support to alleviate the physical and mental burdens of caregiving. However, few studies in China have examined such interventions, and most lack theoretical framework support and standardization. The following key gaps remain in existing caregiver support research. First, the majority of current studies only address caregivers of individuals with dementia only [57,58] or diabetes only [59,60], while interventions for dementia-T2DM co-morbidities caregivers remain scarce. Second, less than 30% of digital intervention studies have articulated their theoretical underpinnings, limiting reproducibility [61,62]. Third, systematic reviews have found incomplete reporting of the implementation process of home and community-based interventions in existing studies (only 28% of studies reported on implementation outcomes) [63], which limits the generalized use of interventions. Fourth, there are fewer relevant studies in China, and most lack theoretical framework support and standardized protocols.

Roy’s Adaptation Model and Fitch’s Theory of Supportive Care are widely used theoretical frameworks for constructing care programs. Roy’s Adaptation Model explains how individuals cope with environmental changes through adaptation, focusing on their adaptation level, stimulating factors, and adaptive mechanisms. While this model provides valuable insights into individual coping dynamics, its emphasis on adaptive processes rather than structured care delivery makes it less suitable for guiding standardized intervention design. In contrast, Fitch’s Theory of Supportive Care addresses and optimizes patients’ physiological, psychological, social, and environmental care needs, emphasizing the importance of personalized care planning, which is more appropriate for this study. Since its inception, this theory has been extensively applied in research addressing supportive care needs for patients and FCGs both domestically and internationally [64-68], yielding notable outcomes. Traditional intervention programs often rely on in-person or telephone formats, focusing mainly on health education while neglecting supportive care needs. Additionally, limited human resources in primary health care services constrain traditional offline interventions, creating significant implementation challenges. While some research platforms have explored online interventions, these often lack transparency in service fees and operate on a small scale, limiting their generalization and promotion. In this context, mHealth supportive care programs offer unique advantages by overcoming the resource and implementation challenges of traditional interventions and providing a novel pathway to meet supportive care needs with promising development prospects.

Building upon Fitch’s Theory of Supportive Care [69] and evidence from cross-sectional surveys, we developed an integrated online-offline mHealth supportive care program targeting FCGs of individuals with comorbid dementia and diabetes in urban-rural grassroots communities. This study aims to clarify the intervention effects of the mHealth supportive care program, with the primary outcome being caregiver burden measured by the Caregiver Burden Inventory (CBI), and secondary outcomes including social support assessed by the Social Support Rating Scale (SSRS) and dementia care knowledge measured by the Dementia Care Knowledge Scale (DCKS). Additionally, subgroup analyses were conducted to evaluate potential heterogeneity of intervention effects across different demographic characteristics such as gender, age, careers, and monthly family income. We hypothesize that, at 3-month follow-up, (a) FCGs receiving the mHealth intervention will demonstrate a significantly greater reduction in CBI scores compared with routine-care controls, and (b) the intervention group will show significantly larger improvements in SSRS and DCKS scores; furthermore, (c) intervention effects may differ across demographic subgroups. Findings from this study will provide evidence for scalable supportive care strategies to address caregiving disparities in resource-limited settings.

## Methods

### Study Design and Objectives

This 2-arm, parallel-group randomized controlled trial evaluated the efficacy of an mHealth supportive care program for FCGs of individuals with comorbid dementia and diabetes. The trial was designed and reported in accordance with the CONSORT (Consolidated Standards of Reporting Trials) 2025 statement [70] (see Checklist 1) and its extension for reporting trials of electronic and mobile health applications (CONSORT-EHEALTH [Consolidated Standards of Reporting Trials of Electronic and Mobile Health Applications and Online Telehealth]) [71] (see Checklist 2).

### Sample Size

#### Simple Size Calculation

The sample size was calculated based on a 1:1 ratio between the intervention and control groups, using the sample size calculation method for comparing the means of two samples. The calculation formula is as follows:$n_{1}=n_{2}=2\left[\frac{\left(\mu_{\alpha }+\mu_{\beta }\right)}{\delta /\sigma }\right]+{\frac{1}{4}}^{2}\mu_{\alpha }{\;}^{2}$

Based on a literature review, the caregiver burden was selected as the primary outcome measure for this study. n_1_ and n_2_ represent the required sample sizes for the two groups, respectively. Due to the lack of prior data from identical designs, the sample size calculation was based on the SD (σ=6.85) and clinically important difference (*δ*=6.87) reported in a quasi-experimental study [72], which tested a dementia caregiver intervention under comparable conditions. Power analysis (*α*=.05, *β*=.1, 2-tailed) indicated 22 participants per group. During the recruitment process, participants showed great enthusiasm in signing up, with the number of applicants exceeding the actual required number by 16. In consideration of ethical principles and the fact that a larger sample size would enhance reliability, we ultimately enrolled 30 per group (total N=60) to further strengthen robustness. Given the exploratory nature of this study, the sample size was estimated based on prior effect sizes, but statistical power may still be limited.

#### Participants

##### Participate Enrollment

This study was conducted at a community health center in Xiamen, Fujian Province, from September 2022 to January 2023. Participants were recruited from 5 urban neighborhood committees and 10 rural villages under the jurisdiction of the selected health center to ensure urban-rural diversity. Due to COVID-19 pandemic restrictions and limited research resources, a single community health center was chosen as the study site.

##### Inclusion Criteria

The inclusion criteria for care recipients are as follows:

- meeting the diagnostic criteria for dementia
- meeting the diagnostic criteria for type 2 diabetes
- aged ≥60 years and permanent resident in the study area

The inclusion criteria for caregivers are as follows:

- legally related to the patient (spouse, child, or sibling), excluding paid caregivers
- providing care ≥8 hours per day, ≥5 days per week for ≥1 month [51]
- conscious adult with basic literacy and comprehension skills
- owning a smartphone with internet access and proficiency in its use
- willing to provide informed consent and participate voluntarily

Providing care ≥8 hours/day, ≥5 days/week for ≥1 month [51]

##### Exclusion Criteria

Patients or caregivers meeting any of the following conditions were excluded:

- having severe physical illness, major diseases, or terminal conditions
- having substance or alcohol dependence
- currently participating in similar intervention studies

##### Withdrawal Criteria

Participation would be terminated in the following conditions:

- if the care recipient was hospitalized for severe illness or passed away
- if the participant voluntarily withdrew from the study for any reason

### Randomization, Allocation Concealment, and Blinding

Random allocation was set at a 1:1 ratio. Participants who were ultimately enrolled in the study were numbered in the order of enrollment. A digital custodian, who did not directly participate in this study, used a computer to generate a random number sequence from 1 to 60. The custodian placed each random number in sequentially numbered, opaque envelopes, which were then sealed and stored to reduce selection and information bias. Researchers inquired about the group assignment of participants by calling the digital custodian. The custodian opened the envelopes in numerical order and informed the researchers of the group assignments. Participants with random numbers 1‐30 were assigned to the intervention group, and those with 31‐60 were assigned to the control group. Since this study was based on an mHealth-supported care intervention, blinding the intervention implementers and participants was challenging. Therefore, blinding was only implemented for data collectors and statistical analysts.

### Interventions

All routine care for patients in both groups was independently performed by FCGs. The control group received offline basic health services and health education, whereas the intervention group received an mHealth supportive care program in addition to the services provided to the control group. The intervention was developed and reported following the TIDieR (Template for Intervention Description and Replication) checklist (see Checklist 3).

Following standard community geriatric chronic disease management protocols, control group participants received monthly home visits (1 hour per session for 12 wk) where implementers delivered targeted health education and addressed caregiver-initiated queries regarding dementia-diabetes comorbidity management. The educational program comprised the following: (a) Diabetes management training: systematic instruction on essential care components, including nutritional regulation, sleep optimization, and exercise regimens; pharmacological guidance included drug categorization, therapeutic mechanisms, dosage administration, and compliance enhancement techniques; training on glycemic monitoring protocols focused on accurate fasting and 2-hour postprandial blood glucose assessment; instruction included early detection of hypoglycemic episodes, ophthalmologic complication signs, crisis management, and dermatological protection measures. (b) Dementia management training: Comprehensive instruction on basic caregiving competencies, including nutritional support, sleep cycle regulation, and physical activity facilitation; educational content encompassed dementia etiology, prognostic trajectories, stage-dependent symptom management, and prevention protocols for elopement, falls, and scald injuries.

**Figure 1.**
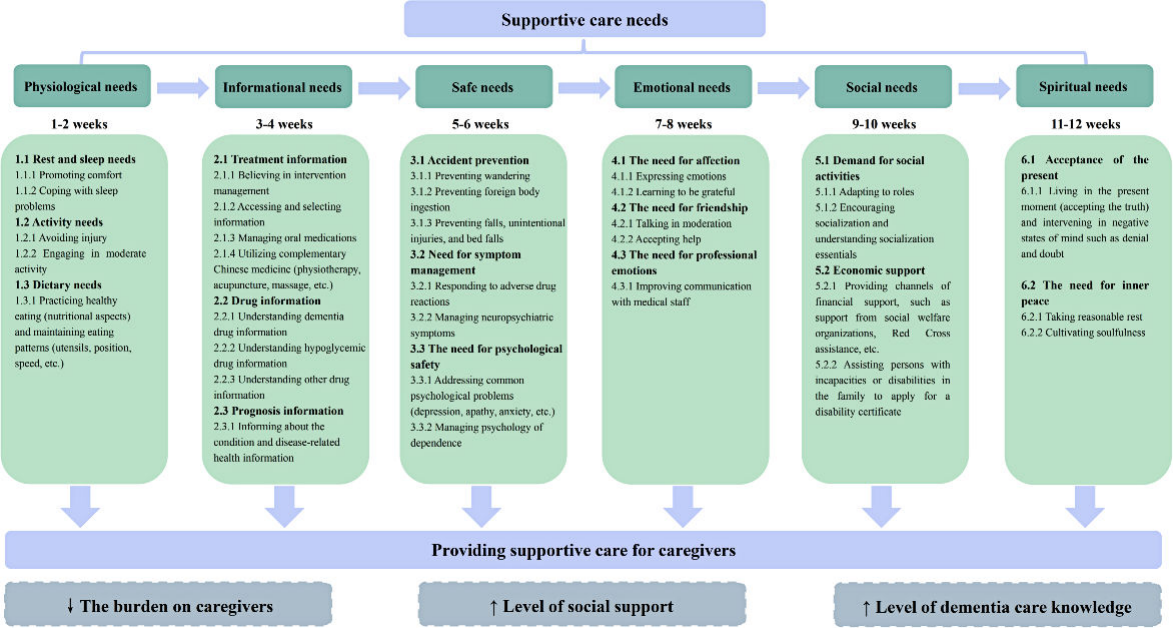
The mHealth supportive care program based on Fitch’s supportive care model for caregivers of patients with dementia and type 2 diabetes: design and intervention components.

The mHealth supportive care intervention was developed based on Fitch’s theoretical model [69], which systematically addresses caregivers’ multidimensional needs. The intervention’s theoretical structure is illustrated in Figure 1; the 6 core intervention modules directly operationalize Fitch’s domains. This intervention program was further informed by baseline epidemiological surveys quantifying caregiving burdens, needs analyses of FCGs, and evidence synthesis through a systematic literature review and Delphi expert consensus. Implementation utilized a robust, municipally administered public health mobile infrastructure to guarantee program fidelity and continuity. The intervention group accessed supplementary weekly mHealth interventions through the “Xiamen i-Health” portal (12 structured sessions across 12 weeks with fortnightly module updates), complemented by asynchronous consultation capabilities. Preintervention preparations and specific steps for implementation are documented in Multimedia Appendix 1. The implementation team comprised professionals with decade-long gerontological nursing practice and 6-year specialized intervention management tenure. Rigorous centralized training was conducted to maintain intervention fidelity, mitigate operational variability, and standardize delivery protocols.

### Data Collection and Outcome Measures

#### Self-Designed Questionnaire

The self-designed questionnaire, developed based on the research objectives and a review of relevant literature [64,72-74], gathered demographic and clinical information, including age, gender, careers, educational levels, monthly family income, living with patient, relationship with patients, as well as current medical history and family history. The questionnaire was evaluated and refined by an expert panel of 5 clinical nursing specialists, all with more than a decade of experience.

#### Primary Outcome

The CBI was used to evaluate the burden experienced by the FCGs of individuals with dementia and diabetes. Zhang [75] adapted this scale from the Chinese version of the CBI developed by Taiwanese scholar Chen et al. [73], with modifications to suit the linguistic characteristics of mainland China. Each item is scored using a Likert 5-point scale ranging from strongly agree (4 points) to strongly disagree (0 points). The total score of the scale ranges from 0 to 96, with higher scores indicating a heavier caregiver burden. Scores ≥24 suggest the need for therapeutic intervention, whereas scores ≥36 indicate a risk of severe burden. The overall Cronbach α coefficient for this scale [75] is 0.77, with an average content validity index (CVI) of 0.95 per item, indicating good reliability and validity. It has been widely used in similar studies [76,77].

#### Secondary Outcomes

The SSRS is a 10-item rating scale that evaluates the psychological support an individual receives in social life and their utilization of such support. The Chinese scholar Shuiyuan [78] developed this scale based on the sociocultural context and familial structure characteristic of Chinese society, ensuring its relevance and validity for use in Chinese populations. The objective support score is the sum of items 2, 4, and 6; the subjective support score is the sum of items 1, 3, 4, and 5; and utilization of support score is the sum of items 8, 9, and 10. Generally, scores below 20 indicate low social support, 20‐30 indicate moderate social support, and 30‐40 indicate satisfactory social support. The test-retest reliability of this scale [78] is 0.92, with item consistency ranging from 0.89 to 0.94 and a Cronbach α coefficient of 0.89. The scale demonstrates good reliability and validity, accurately reflecting an individual’s level of social support. It has been widely used in similar studies [79,80].

The DCKS was used to assess the FCGs’ extent of knowledge about dementia caring [81]. This questionnaire, based on Maslow’s hierarchy of needs, covers personal hygiene, diet, excretion, sleep, disease and rehabilitation, medication, environmental and travel safety, social relationships and family ties, self-esteem, and self-actualization. A Likert scale was used, with 1 point for a correct answer and 0 points for an incorrect answer or “don’t know.” The DCKS scores range from 0 to 22, with higher scores reflecting a higher level of dementia care knowledge. The Cronbach α coefficient for the overall scale [81] is 0.626, with CVI ranging from 0.86 to 1 and an average CVI index of 0.95. It has been widely used in similar studies [82].

#### Adverse Events Monitoring

As this trial evaluated a low-risk supportive care intervention, no systematic, active surveillance for specific adverse events was prespecified. However, any unintended effects or concerns raised by participants during home visits or via the mHealth platform were documented. Furthermore, all instances of participant withdrawal were tracked and reviewed for any potential link to the intervention.

### Statistical Analysis

Data analysis was conducted using IBM SPSS Statistics for Windows version 25.0. Quantitative data with a normal distribution were presented as mean (SD), whereas nonnormally distributed data were presented as median with IQR. Categorical data were presented as frequency and percentage. For comparisons between 2 independent samples, the independent-sample *t* test was used for normally distributed data, whereas the Mann-Whitney *U* test was used for nonnormally distributed data. Comparisons pre- and post-intervention were conducted using a paired *t* test for normally distributed differences or Wilcoxon signed-rank test for nondistributed differences. The *χ*² test or Fisher exact test was used for comparisons of categorical data between groups, and rank-sum tests were used for comparisons of ordinal data. Effect sizes were calculated as Cohen *d* (continuous) or rank correlation r (nonparametric), with 95% CIs reported where applicable. Given the 2-time-point design of this pilot RCT and the completeness of the per-protocol dataset, repeated measures ANOVA was selected as the primary framework for testing group × time interactions and the critical 3-way (group × time × subgroup) interactions in preplanned subgroup analyses. Where significant interactions were detected (*P*<.05), simple effects analyses were performed. The dataset for the final per-protocol analysis was complete, with no missing data at the item level for any outcome measures among participants who completed the follow-up.

### Ethical Considerations

This study was approved by the Ethics Committee of Fujian Provincial Hospital (approval K2021-05-024). Due to delays in the institutional ethics approval process during the COVID-19 pandemic, this trial was retrospectively registered. The trial is registered at ClinicalTrials.gov (NCT06785857; Date: Jan 21, 2025). No changes were made to the trial methods or outcomes after the trial commenced.

Prior to enrollment, all participating caregivers provided written informed consent after being fully informed of the study objectives, procedures, and their right to withdraw at any time without penalty. To ensure confidentiality, all collected data were deidentified and stored securely, with access limited to authorized researchers only. Although no monetary compensation was offered, small gifts (soap, tissue, shopping bags) valued at approximately 20 RMB (equivalent to approximately US $2.97 based on the average exchange rate during the intervention period in 2022) were provided as tokens of appreciation during home visits. We further confirm that no identifiable images, audio, video, or text of participants are included in this manuscript or its supplementary materials. No deviations from the prespecified trial protocol occurred after the commencement of the study.

No patients or members of the public were involved in the design, conduct, reporting, or dissemination of this research.

## Results

### Participants

**Figure 2.**
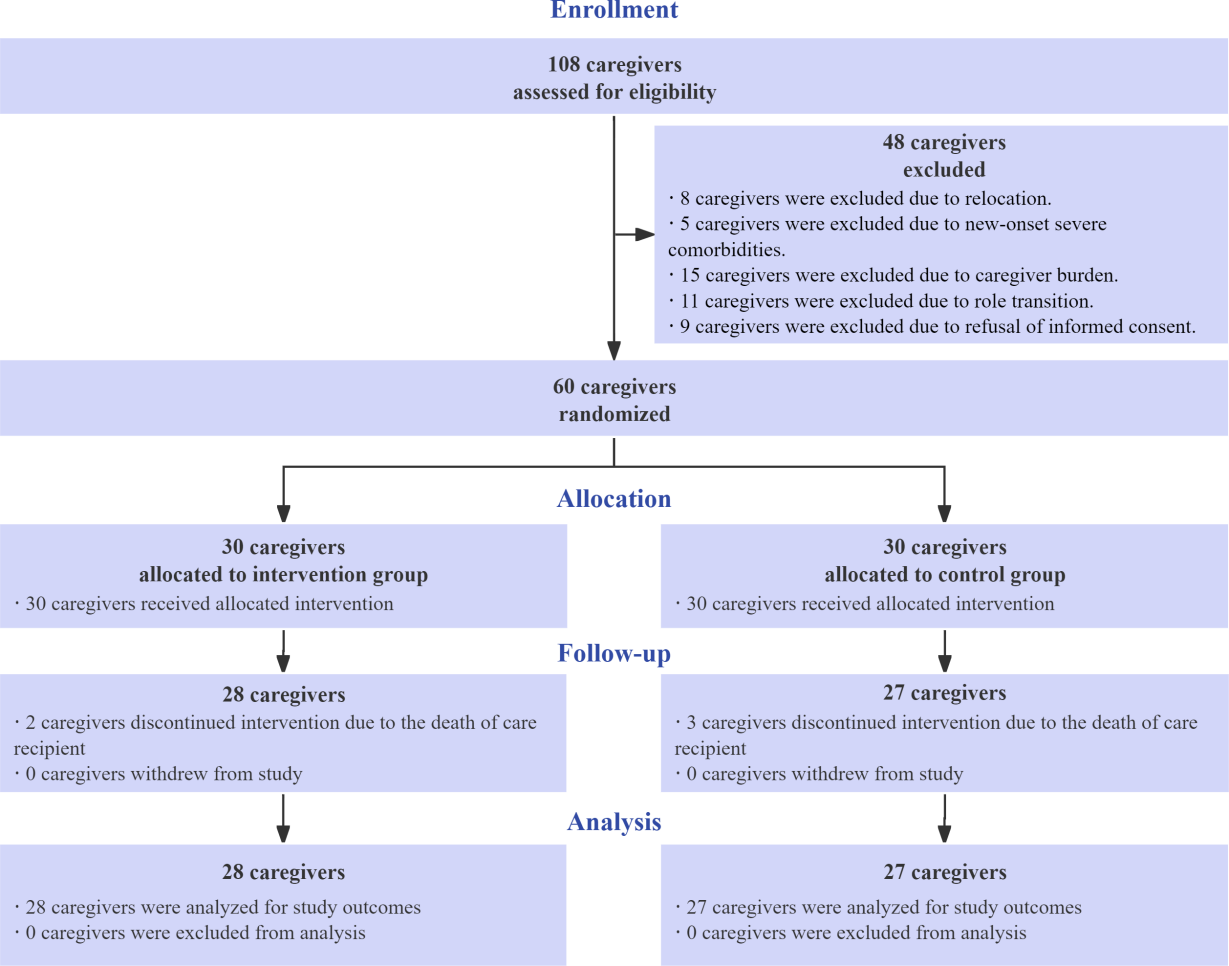
Consolidated Standards of Reporting Trials (CONSORT) flow diagram of participant enrollment, allocation, follow-up, and analysis in a 2-arm, parallel-group, pilot randomized controlled trial evaluating an mHealth supportive care program for family caregivers of individuals with dementia and type 2 diabetes in Xiamen, China (September 2022 to January 2023).

A total of 108 potential participants were assessed for eligibility in this study. Out of the 60 who were eligible, 48 were excluded due to scheduling conflicts and a lack of interest. Consequently, 60 individuals agreed to participate and were randomly assigned to either the intervention group (n=30) or the control group (n=30) after providing informed consent. Herein, due to the death of care recipients during intervention, the number of remaining participants was 28 in the intervention group and 27 in the control group. Ultimately, a total of 55 out of 60 (91.67%) participants completed the follow-up assessments (Figure 2).

### Demographic and Baseline Characteristics

Table 1 presents the sociodemographic characteristics of the caregivers. No significant differences were observed in the characteristics between the intervention and control groups (*P*>.05), indicating that no significant differences between the groups at baseline. Over half (27/55, 49.09%) of the FCGs were aged ≥60. Most caregivers were married (47/55, 85.45%), and 72.73% (40/55) had completed education at the junior high school and above level. Notably, 90.91% (50/55) of caregivers lived with the patients, and 76.36% (42/55) were the patients’ children.

**Table 1.** Comparison of basic demographic information of two groups of caregivers in a pilot randomized controlled trial: caregivers of patients with dementia and type 2 diabetes in Xiamen, China, 2022‐2023.

Items and categories	Control group, n (%)	Intervention group, n (%)	Chi-square (*df*)	*P* value
Gender			0.022 (1)	.88^a^
Male	12 (44.4)	13 (46.4)		
Female	15 (55.6)	15 (53.6)		
Age (y)			1.480 (1)	.22^a^
<60	11 (40.74)	16 (57.14)		
≥60	16 (59.26)	12 (42.86)		
Educational level			1.438 (1)	.16^b^
Primary and below	9 (33.33)	6 (21.43)		
Junior high school and above	18 (66.67)	22 (78.57)		
Marriage			<0.001 (1)	≥.99^a^
Married	23 (85.19)	24 (88.89)		
Unmarried, divorced, widowed, etc	4 (14.81)	4 (14.81)		
Careers			0.379 (1)	0.71^b^
Physical labor	20 (29.63)	20 (32.14)		
Mental labor	7 (14.82)	8 (17.86)		
Living with patient			0.262 (1)	.61^b^
Yes	24 (88.89)	26 (92.86)		
No	3 (11.11)	2 (7.14)		
Relationship with patients			0.059 (1)	.81^a^
Spouse	6 (22.22)	7 (25)		
Children	21 (78.78)	21 (75)		
Monthly family income (RMB)^c^			0.165 (2)	.92^b^
1000‐3000	6 (22.22)	5 (17.86)		
3000‐5000	9 (33.33)	10 (35.71)		
>5000	12 (44.45)	13 (46.43)		

a Refers to the *χ*2 test.

b Refers to Fisher exact test.

c All income values are in RMB. Based on the 2022 exchange rate (US $1≈6.731 RMB), 1,000–3,000 RMB≈US $149–446, 3,000–5,000 RMB≈US $446–743, and >5,000 RMB≈US $>743.

### Comparison of Outcome Measures Pre-intervention

The primary outcome measure in this study was caregiver burden, assessed using the CBI. Secondary outcome measures included caregiver social support level and knowledge level, which were evaluated using the SSRS and DCKS, respectively. Preintervention, no significant differences were detected between the intervention and control groups in CBI (*z*=−0.675, *P*=.50), SSRS (*t*_53_=−0.949, *P*=.35), and DCKS (*t*_53_=−1.256, *P*=.22), indicating that the groups were not statistically different (Table 2).

**Table 2.** Comparison of outcome measures (pre-intervention) between two groups of caregivers in a randomized controlled trial of mHealth intervention for caregivers of patients with dementia and type 2 diabetes in Xiamen, China, 2022‐2023.

Items	Total (N=55)	Control group (n=27)	Intervention group (n=28)	Statistic	*P* value	Cohen *d*/*r*
SSRS^a^, median (IQR)	24.00 (22.00 to 26.00)	24.00 (22.00 to 26.00)	25.00 (23.25 to 26.00)	−0.949 (53)^b^	.35^c^	Cohen *d*=0.26 (−0.28 to 0.79)
CBI^d^, median (IQR)	57.00 (53.00 to 60.00)	56.00 (53.00 to 60.00)	57.00 (54.00 to 60.75)	−0.675^e^	.50^f^	*r*=0.09 (0.01 to 0.38)
DCKS^g^, median (IQR)	11.00 (10.00 to 13.00)	12.00 (10.00 to 16.00)	11.00 (10.00 to 12.75)	−1.256 (53)^b^	.22^c^	Cohen *d*=0.34 (−0.19 to 0.87)

a SSRS: Social Support Rating Scale.

b *t* test value (*df*)

c Normally distributed variables are presented as mean (SD) (independent *t* test).

d CBI: Caregiver Burden Inventory.

e *z* score.

f Nonnormal variables are represented as median (IQR) (Mann-Whitney *U* test).

g DCKS: Dementia Caring Knowledge Scale.

### Comparison of Outcome Measures Post-intervention

Postintervention, significant differences were observed between the intervention and control groups in SSRS (*z*=2.494, *P*=.01), CBI (*z*=−3.534, *P*<.001), and DCKS scores (*z*=−4.233, *P*<.001). The results are presented in Table 3.

**Table 3.** Comparison of outcome measures (postintervention) between two groups of caregivers in a randomized controlled trial of mHealth intervention for caregivers of patients with dementia and type 2 diabetes in Xiamen, China, 2022‐2023.

Items	Total (N=55)	Control group (n=27)	Intervention group (n=28)	*z*	*P* value	*r*
SSRS^a^, median (IQR)	31.00 (29.00-33.00)	31.00 (28.00-32.00)	32.00 (30.00-34.00)	2.494	.01^b^	0.34 (0.09-0.55)
CBI^c^, median (IQR)	50.00 (48.00-52.00)	52.00 (49.00-55.00)	48.00 (46.00-50.75)	−3.534	<.001^b^	0.48 (0.24-0.68)
DCKS^d^, median (IQR)	18.00 (16.00-19.00)	16.00 (15.00-18.00)	19.00 (18.00-19.00)	−4.233	<.001^b^	0.57 (0.35-0.75)

a SSRS: Social Support Rating Scale.

b Nonnormal variables are represented as median (IQR) (Mann-Whitney *U* test).

c CBI: Caregiver Burden Inventory.

d DCKS: Dementia Caring Knowledge Scale.

### Comparison of Outcome Measures in the Intervention Group Pre- and Post-intervention

Within the intervention group, statistically significant differences were observed in SSRS (*t*_27_=−14.959, *P*<.001), CBI (*t*_27_=11.415, *P*<.001), and DCKS scores (*z*=4.637, *P*<.001) pre- and postintervention (Table 4).

**Table 4.** Comparison of outcome measures (pre- and post-intervention) in the intervention group of a randomized controlled trial of mHealth intervention for caregivers of patients with dementia and type 2 diabetes in Xiamen, China, 2022‐2023.

Items	Preintervention (n=28)	Postintervention (n=28)	Statistic	*P* value	Cohen *d*/*r*
SSRS^a^, median (IQR)	25.00 (23.25-26.00)	32.00 (30.00-34.00)	−14.959^b^(27)	<.001^c^	Cohen *d*=2.83 (1.99-3.66)
CBI^d^, median (IQR)	57.00 (54.00-60.75)	48.00 (46.00-50.75)	11.415^b^(27)	<.001^c^	Cohen *d*=2.16 (1.47-2.83)
DCKS^e^, median (IQR)	11.00 (10.00-12.75)	19.00 (18.00-19.00)	4.637^f^	<.001^g^	*r*=0.86^h^

a SSRS: Social Support Rating Scale.

b *t* test value (*df*).

c Normally distributed variables are presented as mean (SD) (paired *t* test).

d CBI: Caregiver Burden Inventory.

e DCKS: Dementia Caring Knowledge Scale.

f *z* score.

g Nonnormal variables are represented as median (IQR) (Mann-Whitney *U* test).

h Regarding the Wilcoxon signed-rank test, there is currently no consensus in the statistical literature on a standardized method for calculating confidence intervals for its effect size (eg, *r*), and commonly used statistical software (eg, SPSS, R) does not provide this functionality.

### Subgroup Analysis of Intervention Effects by Caregiver Characteristics

The 3-way interaction analyses indicated that changes in SSRS and DCKS scores were not significantly moderated by the examined demographic variables (all 3-way interactions *P*>.05; see Multimedia Appendix 2). For CBI score changes, 3-way interaction tests revealed significant time×group×gender (*F*_1,51_=6.712, *P*=.01), time×group×age (*F*_1,51_=7.784, *P*=.007), time×group×careers (*F*_1,51_=8.327, *P*=.006), time×group×educational level (*F*_1,51_=8.325, *P*=.006), and time×group×monthly family income (*F*_1,51_=3.793, *P*=.03); therefore, subgroup analyses were conducted by sex, age, careers, educational level, and monthly family income.

As shown in Table 5, the gender subgroup analysis indicated that male participants in the control group had significantly higher CBI scores postintervention compared to those in the intervention group (*t*_23_*=*5.289*, P*=.01), whereas no significant differences were observed between the groups for females (*t*_28_=0.427, *P*=.59). In the age subgroup analysis, caregivers under 60 years in the control group exhibited higher CBI scores (*t*_25_=4.088, *P*=.02), with no differences found among those aged 60 years and older (*t*_26_=0.363, *P*=.64). The careers subgroup analysis revealed that participants engaged in physical labor had higher scores in the control group (*t*_38_=4.249, *P*=.02), while no differences were noted for those in mental labor (*t*_13_=0.208, *P*=.72). Regarding the educational level subgroup analysis, caregivers with primary education or below in the control group showed significantly higher CBI scores post-intervention (*t*_13_=4.494, *P*=.04), whereas no significant differences were observed among those with junior high school education and above (*t*_38_=1.273, *P*=.16). In the monthly family income subgroup, caregivers with incomes between 1000 and 3000 RMB (approximately US $149-446) showed a marginally significant trend toward higher scores in the control group (*t*_9_=6.422, *P*=.06), and no statistical differences were observed in the higher income groups.

**Table 5.** Subgroup analysis of intervention effects on CBI^a^ by caregiver characteristics in a randomized controlled trial of mHealth intervention for caregivers of patients with dementia and type 2 diabetes in Xiamen, China, 2022‐2023.

Variable, categories, and time	Mean difference (SE; 95% CI)	*t* value	*P *value
Gender			
Male			
Preintervention	−1.99 (2.834; −7.68 to 3.70)	−1.139 (23)	.49
Postintervention	5.04 (1.889; 1.25 to 8.83)	5.289 (23)	.01
Female			
Preintervention	−0.80 (2.585; −5.99 to 4.39)	−0.252 (28)	.76
Postintervention	0.93 (1.723; −2.53 to 4.39)	0.427 (28)	.59
Age (y)			
<60			
Preintervention	−2.93 (2.751; −8.46 to 2.59)	−1.676 (25)	.29
Postintervention	4.32 (1.856; 0.59 to 8.04)	4.088 (25)	.02
≥60			
Preintervention	−0.73 (2.713; −6.18 to 4.72)	−0.214 (26)	.78
Postintervention	0.85 (1.830; −2.82 to 4.53)	0.363 (26)	.64
Careers			
Physical labor			
Preintervention	−2.36 (2.266; −6.91 to 2.19)	−1.543 (38)	.30
Postintervention	3.60 (1.516; 0.56 to 6.64)	4.249 (38)	.02
Mental labor			
Preintervention	1.57 (3.704; −5.87 to 9.01)	0.264 (13)	.67
Postintervention	0.89 (2.478; −4.08 to 5.87)	0.208 (13)	.72
Educational level			
Primary and below			
Preintervention	-0.36 (3.920; -11.47 to 4.27)	−1.516 (13)	.36
Postintervention	5.50 (2.646; 0.19 to 10.81)	4.494 (13)	.04
Junior high school and above			
Preintervention	-0.03 (2.274; -4.59 to 4.54)	−0.010 (38)	.99
Postintervention	2.20 (1.536; -0.88 to 5.29)	1.273 (38)	.16
Monthly family income (RMB)^b^			
1000‐3000			
Preintervention	−3.18 (4.559; 12.34 to 5.98)	−1.209 (9)	.49
Postintervention	6.00 (3.048; 0.163 to 12.13)	6.422 (9)	.06
3000‐5000			
Preintervention	−1.56 (3.342; −8.27 to 5.16)	−0.700 (17)	.64
Postintervention	3.40 (2.234; −1.09 to 7.89)	2.365 (17)	.13
>5000			
Preintervention	−0.37 (2.912; −6.22 to 5.49)	−0.097 (23)	.90
Postintervention	1.12 (1.947; −2.79 to 5.03)	0.431 (23)	.57

a CBI: Caregiver Burden Inventory.

b All income values are in RMB. Based on the 2022 exchange rate (1 US $≈6.731 RMB), 1,000–3,000 RMB≈US $149–446, 3,000–5,000 RMB≈US $446–743, and >5,000 RMB≈>US $743.

### Adverse Events

No serious adverse events or intervention-related harms were reported among caregivers in either group during the trial. All participant withdrawals (2 in the intervention group and 3 in the control group) were attributable to the death of their care recipients. No predefined adverse outcomes or deaths occurred among the caregiver participants.

## Discussion

### Principal Results

This study evaluated an mHealth supportive care program based on Fitch’s theoretical framework for community-recruited caregivers of people with dementia and diabetes. Screening, recruitment, and participation outcomes indicated that although the number of participants was limited, the vast majority were willing to participate and complete the program. Over the 12-week intervention period, under the supervision and guidance of community health workers, the program exerted positive effects on FCGs of individuals with dementia and diabetes. Specifically, the intervention group demonstrated a statistically significant and substantial reduction in caregiver burden, along with considerable improvements in dementia care knowledge and perceived social support. These robust findings align with existing literature [83,84], supporting the efficacy of structured digital health interventions in alleviating caregiver burden while enhancing caregiving knowledge and perceived support.

### Comparison With Prior Work

The observed intervention effects can be attributed to the program’s systematic approach to meeting the multidimensional needs of FCGs. The significant reduction in caregiver burden is consistent with the Stress Process Model [85], which suggests that interventions enhancing caregivers’ coping resources can alleviate their burden. The platform facilitated health care professionals’ delivery of accurate, comprehensible information to FCGs, potentially increasing caregivers’ confidence in dementia management, which has a positive effect on reducing caregiver burden [86]. Furthermore, the emotional, social, and spiritual needs module also encouraged caregivers to seek help from family, friends, and health care professionals, activating social support networks and thereby alleviating stress. This buffering effect aligns with the social support model [87], emphasizing the role of social networks in reducing caregiving stress. It is noteworthy that, despite a clinically meaningful reduction (approximately 15%), postintervention burden scores remained above the high-threshold level (36). This underscores the persistent, severe strain inherent in dementia caregiving [88] and highlights that while mHealth support can provide significant relief, it may need to be part of longer-term, adaptive, or multicomponent strategies to address profound, chronic burden fully.

The significant improvement in social support scores within the intervention group indicates the program’s efficacy in enhancing caregivers’ perception and utilization of social support, aligning with the findings of Tremont et al [89], which underscores the importance of strengthening social support for caregivers. The mechanism of action for this change is consistent with the social support buffer model [87]. On the one hand, the design of the social demand module not only facilitates communication between FCGs and health care workers to reduce social isolation [90] but also provides practical advice for FCGs to cope with stressors and improve their social support capacity. On the other hand, the online platform provided FCGs facing stigmatization, social isolation, or resource deprivation with key alternative pathways for emotional recognition and practical advice, as evidenced in other stigmatized health domains [91-94]. This dual-support model proved effective in alleviating negative emotions such as loneliness and helplessness [95,96], thereby improving caregivers’ quality of life [97,98].

Acquiring relevant knowledge is a prerequisite and foundation for improving skill levels [99]. The median dementia care knowledge score in the intervention group increased significantly from 11 to 19, with a significant between-group difference also observed. This was reflected in caregivers’ enhanced ability to recognize early complications more accurately, perform more standardized medication management (eg, avoiding incorrect and missed doses), and use nonpharmacological intervention techniques to address common behavioral symptoms. This design aligns with the “goal-oriented” principle of adult learning by providing learners with a clear modular knowledge structure. This improvement (approximately 73%) far exceeded the 20% to 30% knowledge gain achieved by home care workers through online training reported in the studies by Yeh et al [100,101], demonstrating the effectiveness of the mHealth supportive care program designed based on the supportive care framework in this study. This improvement can be attributed to the combination of online learning modules, hands-on demonstration videos, and offline follow-up coaching, which together reinforced caregivers’ competence and confidence in managing dementia care [102,103].

The differential intervention effects on caregiver burden across demographic subgroups are a critical finding. Greater burden reduction among male, younger (<60 y), manual labor, caregivers with primary education or below, and lower-income caregivers suggests that the mHealth program’s value is particularly pronounced for those who may face barriers to traditional support. This aligns with literature noting higher technology acceptance among males [104,105] and the greater reliance on accessible, free resources among economically disadvantaged groups [106-109], and the finding that individuals with lower educational attainment, who often have limited access to diverse health information sources [110,111], may rely more on structured digital tools that lower the barrier to information acquisition [112]. Conversely, the attenuated effects observed among female caregivers may stem from a misalignment between the intervention’s primarily informational and instrumental support and their often more pronounced needs for emotional coping strategies and skills to manage role conflict and intra-family dynamics [113-115]. The text-based, asynchronous nature of the mHealth platform might have been less effective in addressing these immediate emotional and relational stressors. For older caregivers (≥60 y), lower digital literacy and potential discomfort with mobile technology [116,117] could have hindered full engagement with the digital modules, thereby diluting the intervention’s potential impact [118]. Among knowledge workers and caregivers with higher educational attainment (junior high school and above), their preexisting proficiency in information-seeking and likely higher access to alternative educational resources may have diminished the marginal benefit provided by our structured program. Similarly, higher-income caregivers likely have established access to private care services or consultations [119], making a supplementary public mHealth program less critical for burden alleviation. Importantly, the consistent improvements in social support and knowledge across all subgroups confirm that the program’s core educational and connective functions delivered universal value, irrespective of these moderating factors for burden reduction.

### Limitations

This study has several limitations that warrant consideration. First, although the study achieved statistical power, the geographical restriction to a single urban-rural cluster in Xiamen (5 neighborhood committees and 10 villages) may limit the generalizability of the findings. Additionally, the relatively small sample size could further constrain the population representativeness. Second, this study did not evaluate the cost-effectiveness of the mHealth supportive care program, which is critical for real-world implementation. Third, the COVID-19 pandemic disrupted in-person follow-ups, necessitating alternative methods (eg, phone interviews) that may introduce bias. Then, the DCKS used in this study demonstrated a Cronbach α of 0.626, which, while acceptable for a pilot study, suggests that future large-scale studies should consider using more established scales or further refining this instrument. The scale’s reliability may be influenced by item quantity, cultural adaptation factors, and participant heterogeneity. Finally, although the 12-week intervention duration is consistent with comparable studies [120-124], the intervention period was insufficient to assess long-term effects on patient quality of life or program sustainability.

### Conclusions

This study examined the efficacy of a supportive care framework-based mHealth intervention for FCGs of individuals with dementia and diabetes. Its primary innovation lies in its theory-driven design that addresses the complex, multidimensional needs of this comorbid caregiving population, distinguishing it from interventions targeting single conditions. The program significantly improved caregiver burden, social support, and dementia care knowledge, contributing novel evidence to a sparsely researched area. Importantly, subgroup analyses revealed differential effectiveness across demographic groups, suggesting that mHealth interventions can be tailored to enhance equity in access to support resources. In practical terms, by offering convenient, low-cost services, integrating health care resources, and enhancing personalized education, the program demonstrates the substantial potential of mHealth technology to improve caregiving outcomes. Furthermore, integrating structured mHealth support with existing community health resources presents a viable and scalable strategy to alleviate caregiver burden in resource-constrained settings. This approach not only helps FCGs cope with practical challenges but also provides a new perspective for promoting health equity at the grassroots level. Future research and implementation should incorporate adaptive or tailored features to meet diverse caregiver needs and advance health equity. Subsequent research should prioritize multicenter trials with longer follow-up, formal cost-effectiveness analyses, and the development of strategies to personalize digital supportive care.

## Supplementary material

10.2196/72012 Multimedia Appendix 1 The process and content of the mHealth supportive care intervention program. mHealth: mobile health.

10.2196/72012 Multimedia Appendix 2 Results of repeated-measures ANOVA for different demographic variables on outcome indicators.

10.2196/72012 Checklist 1 CONSORT 2025 checklist.

10.2196/72012 Checklist 2 CONSORT-EHEALTH checklist (V1.6.1).

10.2196/72012 Checklist 3 The TIDieR checklist.

## Abbreviations

CBI: Caregiver Burden Inventory
CONSORT: Consolidated Standards of Reporting Trials
CONSORT-eHEALTH: Consolidated Standards of Reporting Trials of Electronic and Mobile Health Applications and Online Telehealth
CVI: content validity index
DCKS: Dementia Caring Knowledge Scale
FCG: family caregiver
mHealth: mobile health
SSRS: Social Support Rating Scale
T2DM: type 2 diabetes mellitus
TIDieR: Template for Intervention Description and Replication
