# Supplementary material for: Effects of the mHealth Supportive Care Program for Family Caregivers of Individuals With Dementia and Diabetes: Pilot Randomized Controlled Trial
Source: JMIR Mhealth Uhealth. 2026 Mar 24;14:e72012. doi: 10.2196/72012 (PMC13012235; doi:10.2196/72012)
Supplement: Multimedia Appendix 1 [file mhealth-v14-e72012-s001.docx]

**mHealth Supportive Care Intervention Program Process**

| **Stage** | **Project** | **Element** | **Specifics** |
| --- | --- | --- | --- |
| **mHealth intervention**  **Pre-protocol upfront**  **Preparation** | Offline preparation | Assess caregivers | (1) Basic assessment: relationship with the patient (whether it is a family member), physical condition, consciousness, reading and comprehension ability;  (2) Whether the caregiver is the primary caregiver in terms of hours and frequency of caregivers. |
|  |  | Material | (1) Participants' devices: each caregiver needs to have at least one terminal device, such as a mobile smartphone/tablet;  (2) Make a copy of the informed consent form, and each participating caregiver understands the trial process and signs the informed consent form. |
|  |  | Environmental facilities | (1) Wi-Fi facilities: Researchers and caregivers participating in the intervention trial need to have Wi-Fi facilities;  (2) Offline follow-up intervention activity venues (caregivers' homes and health management rooms of the investigators' workplaces): choose brighter and quieter activity venues. |
|  |  | Personnel preparation | (1) The researcher formed an intervention group with the helpers of the neighborhood committees, grid members and village doctors, who were responsible for contacting the caregivers and notifying the households.  (2) Unified training of data collectors and data entry personnel, responsible for data collection and entry. |

**mHealth Supportive Care Intervention Program Process (continue)**

| **Stage** | **Project** | **Element** | **Specifics** |
| --- | --- | --- | --- |
|  | Prepare online | Mobile health platform management | (1) The caregivers who agree to participate in the trial will download and bind the mobile phone of "Xiamen i Health" to inform them of the use method and precautions, and operate it in real time on the spot until the caregiver truly grasps the use method.  (2) The researcher is bound to the reminder function of "i Health Intelligent Assistant", which is convenient for receiving and replying to online questions and making appointments with experts in a timely manner. |
| **mHealth intervention** | Pre-intervention | The basic content of the mHealth intervention program | The contents of the intervention included: physiological care needs, information needs, safety needs, emotional needs, social needs, and mental needs, literature review, and information editing. |
|  | At the time of intervention | Composition of the Expert Group | Experts in relevant fields will be invited to obtain the consent of experts by telephone or email, and an expert meeting group will be formed to discuss and deliberate the content of the plan. |

**mHealth Supportive Care Intervention Program Process (continue)**

| **Stage** | **Project** | **Element** | **Specifics** |
| --- | --- | --- | --- |
|  |  | The caregiver determines the content, duration, frequency, duration, etc. of the intervention | The content of the intervention consisted of 6 care needs based on the results of the previous current situation survey, and the online intervention cycle was 12 weeks, each content was intervened for 2 weeks, and the intervention was conducted once a week, each time for 1 hour, and the researchers used online tweets as the carrier to push the family caregivers, and the specific content centered on the 6 care needs (see Annex 4), and the caregivers could also consult with the researchers online at any time for relevant care issues and interactive communication. The online intervention, such as the operation of nursing skills, requires on-site demonstrations by caregivers to clarify whether they have mastered the situation, and the researchers conduct follow-up visits at home. |
|  | Post-intervention | Feedback and Q&A | (1) Listen to the feedback of caregivers and answer questions about the care process.  (2) Relevant practical care skills need to be demonstrated on site, and the next home time should be made with the caregiver, which is the key content of offline follow-up. |
|  |  | Make an appointment for the next intervention time | Make an appointment with the caregiver for the next intervention and send the content of the next intervention, so that the caregiver can preview and comprehend the reading in advance. |

**Contents of the mHealth Supportive Care Intervention Program**

| **Project** | **Time** | **Content of the Intervention** |
| --- | --- | --- |
| **1 Physiological Needs** | Weeks 1- 2 | **1.1 Rest and sleep needs**  1.1.1 Promoting comfort  1.1.2 Coping with sleep problems  **1.2 Activity needs**  1.2.1 Avoiding injury  1.2.2 Engaging in moderate activity  **1.3 Dietary needs**  1.3.1 Practicing healthy eating (nutritional aspects) and maintaining eating patterns (utensils, position, speed, etc.) |
| **2 Information Needs** | Weeks 3- 4 | **2.1 Treatment information**  2.1.1 Believing in intervention management  2.1.2 Accessing and selecting information  2.1.3 Managing oral medications  2.1.4 Utilizing complementary Chinese medicine (physiotherapy, acupuncture, massage, etc.)  **2.2 Drug Information**  2.2.1 Understanding dementia drug information  2.2.2 Understanding hypoglycemic drug information  2.2.3 Understanding other drug information  **2.3 Prognosis information**  2.3.1 Informing about the condition and disease-related health information |

**Contents of the mHealth Supportive Care Intervention Program (continue)**

| **Project** | **Time** | **Content of the Intervention** |
| --- | --- | --- |
| **3 Security Needs** | Weeks 5- 6 | **3.1 Accident prevention**  3.1.1 Preventing wandering  3.1.2 Preventing foreign body ingestion  3.1.3 Preventing falls, unintentional injuries, and bed falls  **3.2 Need for symptom management**  3.2.1 Responding to adverse drug reactions  3.2.2 Managing neuropsychiatric symptoms  **3.3 The need for psychological safety**  3.3.1 Addressing common psychological problems (depression, apathy, anxiety, etc.)  3.3.2 Managing psychology of dependence |
| **4 Emotional Needs** | Weeks 7 - 8 | **4.1 The need for affection**  4.1.1 Expressing emotions  4.1.2 Learning to be grateful  **4.2 The Need for Friendship**  4.2.1 Talking in moderation  4.2.2 Accepting help  **4.3 The Need for Professional Emotions**  4.3.1 Improving communication with medical staff |

**Contents of the mHealth Supportive Care Intervention Program (continue)**

| **Project** | **Time** | **Content of the Intervention** |
| --- | --- | --- |
| **5 Social Needs** | Weeks 9 - 10 | **5.1 Demand for social activities**  5.1.1 Adapting to roles  5.1.2 Encouraging socialization and understanding socialization essentials  **5.2 Economic support**  5.2.1 Providing channels of financial support, such as support from social welfare organizations, Red Cross assistance, etc.  5.2.2 Assisting persons with incapacities or disabilities in the family to apply for a disability certificate |
| **6 Spiritual Needs** | Weeks 11- 12 | **6.1 Acceptance of the present**  6.1.1 Living in the present moment (accepting the truth) and intervening in negative states of mind such as denial and doubt  **6.2 The need for inner peace**  6.2.1 Taking reasonable rest  6.2.2 Cultivating soulfulness |
