# Supplementary material for: Effects of the mHealth Supportive Care Program for Family Caregivers of Individuals With Dementia and Diabetes: Pilot Randomized Controlled Trial
Source: JMIR Mhealth Uhealth. 2026 Mar 24;14:e72012. doi: 10.2196/72012 (PMC13012235; doi:10.2196/72012)
Supplement: Multimedia Appendix 2 [file mhealth-v14-e72012-s002.docx]

**Repeated Measures ANOVA of Demographic Variables on CBI Scores**

1. **Main Effect of Time**

The repeated measures ANOVA revealed statistically significant main effects of time across all models (all *P* < 0.001), indicating that both caregiver groups exhibited a significant downward trend in burden levels (CBI scores) from pre-intervention (T1) to post-intervention (T2). These results suggest that the intervention had a universal effect on reducing caregiver burden, irrespective of group allocation or demographic characteristics.

1. **Three-Way Interaction (Time × Group × Variable)**

Triple interaction effect analyses of time **×** subgroup **×** demographic variables revealed significant moderating effects of gender (*F* = 6.712, *P* = 0.012), age (*F* = 7.784, *P* = 0.007), monthly family income (*F* = 3.793, *P* = 0.029), and careers (*F* = 8.327, *P* = 0.006) on the effect of the intervention; education levels (*F* = 8.325, *P* = 0.006), living with patient status (*F* = 0.011, *P* = 0.917), marriage (*F* = 0.096, *P* = 0.758), and relationship with patient (*F* = 3.182, *P* = 0.080) did not show moderating effects. The above results suggest that the effect of the intervention was heterogeneous across caregivers of different genders, ages, education levels, monthly family income, and **careers**.

**Table 1. Repeated Measures ANOVA Results of Demographic Variables on CBI Scores**

| **Variable** | **Between-Group Effect**  ***F*(*P*)** | **Within-Group Effect**  ***F*(*P*)** | **Interaction Effect**  **(Time × Group)**  ***F*(*P*)** | **Interaction Effect**  **(Time × Variable)**  ***F*(*P*)** | **Interaction Effect**  **(Time × Group × Variable)**  ***F*(*P*)** |
| --- | --- | --- | --- | --- | --- |
| Gender | 0.264 (0.610) | 154.213 (<0.001)*** | 18.367 (<0.001)*** | 2.150 (0.149) | 6.712 (0.012)* |
| **Age** | 0.058 (0.811) | 163.870 (<0.001)*** | 18.915 (<0.001)*** | 2.431 (0.125) | 7.784 (0.007)** |
| **Educational level** | 0.307 (0.582) | 105.561 (<0.001)*** | 22.615 (<0.001)*** | 1.072 (0.152) | 8.325 (0.006)** |
| **Living with patient** | 3.978 (0.051) | 36.673 (<0.001)*** | 4.280 (0.044)* | 0.254 (0.616) | 0.011 (0.917) |
| Relationship with patient | 0.319 (0.574) | 102.951 (<0.001)*** | 17.960 (<0.001)*** | 0.136 (0.714) | 3.182 (0.080) |
| **Monthly Family Income (RMB)** | 0.278 (0.600) | 121.757 (<0.001)*** | 21.687 (<0.001)*** | 0.853 (0.432) | 3.793 (0.029)* |
| **Marriage** | 0.203 (0.654) | 64.513 (<0.001)*** | 8.323 (0.006)** | 0.002 (0.965) | 0.096 (0.758) |
| **Careers** | 0.278 (0.600) | 113.468 (<0.001)*** | 5.273 (0.026)* | 0.146 (0.704) | 8.327 (0.006)** |
| Note: Significance levels are denoted as: ****P* < 0.001, ***P* < 0.01, **P* < 0.05. | | | | | |

**Repeated Measures ANOVA of Demographic Variables on SSRS Scores**

1. **Main Effect of Time**

The repeated measures ANOVA revealed statistically significant main effects of time across all models (all *P* < 0.001), indicating that both caregiver groups exhibited a significant downward trend in social support levels (SSRS scores) from pre-intervention (T1) to post-intervention (T2). These results suggest that the intervention had a universal effect on improving social support level, irrespective of group allocation or demographic characteristics.

1. **Three-Way Interaction (Time × Group × Variable)**

Triple interaction effect analyses of time × subgroup × demographic variables revealed no significant moderating variables for all demographic variables. These results suggest that the effect of the intervention on SSRS scores was primarily driven by the interaction of time and subgroups, while the moderating effect of demographic variables was weak overall.

**Table 2. Repeated Measures ANOVA Results of Demographic Variables on SSRS Scores**

| **Variable** | **Between-Group Effect**  ***F*(*P*)** | **Within-Group Effect**  ***F*(*P*)** | **Interaction Effect**  **(Time × Group)**  ***F*(*P*)** | **Interaction Effect**  **(Time × Variable)**  ***F*(*P*)** | **Interaction Effect**  **(Time × Group × Variable)**  ***F*(*P*)** |
| --- | --- | --- | --- | --- | --- |
| Gender | 4.750 (0.034)* | 284.447 (<0.001)*** | 5.168 (0.027)* | 10.357 (0.002)** | 0.704 (0.405) |
| **Age** | 9.812 (0.003)** | 271.571 (<0.001)*** | 3.054 (0.087) | 5.717 (0.021)* | 0.456 (0.502) |
| **Educational level** | 3.940 (0.092) | 169.487 (< 0.001)*** | 9.504 (0.003)** | 11.483 (0.001)** | 2.080 (0.155) |
| **Living with patient** | 0.384 (0.538) | 67.723 (<0.001)*** | 2.127 (0.151) | 0.638 (0.428) | 0.160 (0.691) |
| Relationship with patient | 0.565 (0.456) | 182.777 (<0.001)*** | 9.366 (0.004)** | 20.130 (<0.001)*** | 3.285 (0.076) |
| **Monthly Family Income (RMB)** | 3.102 (0.054) | 228.604 (<0.001)*** | 9.881 (0.003)** | 8.791 (0.001)** | 1.602 (0.212) |
| **Marriage** | 0.296 (0.589) | 129.627 (<0.001)*** | 0.655 (0.422) | 0.140 (0.710) | 0.655 (0.422) |
| **Careers** | 1.654 (0.190) | 236.618 (<0.001)*** | 5.875 (0.019)* | 4.764 (0.006)** | 0.446 (0.722) |
| Note: Significance levels are denoted as: ****P* < 0.001, ***P* < 0.01, **P* < 0.05. | | | | | |

**Repeated Measures ANOVA of Demographic Variables on DCKS Scores**

1. **Main Effect of Time**

The repeated measures ANOVA revealed statistically significant main effects of time across all models (all *P* < 0.001), indicating a trend of significant changes in DCKS scores from pre-intervention (T1) to post-intervention (T2) for both caregiver groups. This result suggests the generalizability of the influence of the time factor on DCKS scores regardless of subgroup or demographic characteristics.

1. **Three-Way Interaction (Time × Group × Variable)**

Triple interaction effect analyses of time × subgroup × demographic variables revealed no significant moderating variables for all demographic variables. These results suggest that the effect of the intervention on DCKS scores was primarily driven by the interaction of time and subgroups, while the moderating effect of demographic variables was weak overall.

**Table 3. Repeated Measures ANOVA Results of Demographic Variables on DCKS Scores**

| **Variable** | **Between-Group Effect**  ***F*(*P*)** | **Within-Group Effect**  ***F*(*P*)** | **Interaction Effect**  **(Time × Group)**  ***F*(*P*)** | **Interaction Effect**  **(Time × Variable)**  ***F*(*P*)** | **Interaction Effect**  **(Time × Group × Variable)**  ***F*(*P*)** |
| --- | --- | --- | --- | --- | --- |
| Gender | 1.456 (0.233) | 435.308 (<0.001)*** | 38.393 (<0.001)*** | 2.662 (0.109) | 1.175 (0.284) |
| **Age** | 1.019 (0.318) | 413.255 (<0.001)*** | 37.642 (<0.001)*** | 1.298 (0.260) | 0.366 (0.548) |
| **Educational level** | 0.862 (0.358) | 322.819 (<0.001)*** | 26.852 (<0.001)*** | 1.605 (0.211) | 0.261 (0.611) |
| **Living with patient** | 0.086 (0.771) | 125.080 (<0.001)*** | 10.211 (0.002)** | 0.040 (0.842) | 0.040 (0.842) |
| Relationship with patient | 2.547 (0.117) | 313.847 (<0.001)*** | 21.877 (<0.001)*** | 0.783 (0.380) | 0.548 (0.462) |
| **Monthly Family Income (RMB)** | 1.562 (0.217) | 387.173 (<0.001)*** | 32.612 (<0.001)*** | 1.595 (0.213) | 1.607 (0.211) |
| **Marriage** | 0.060 (0.808) | 201.216 (<0.001)*** | 26.100 (<0.001)*** | 0.072 (0.790) | 1.481 (0.229) |
| **Careers** | 1.159 (0.287) | 313.297 (<0.001)*** | 23.185 (<0.001)*** | 1.851 (0.151) | 2.090 (0.114) |
| Note: Significance levels are denoted as: ****P* < 0.001, ***P* < 0.01, **P* < 0.05. | | | | | |
